# Sex and age differences of postural control in community-dwelling older adults

**DOI:** 10.3389/fnhum.2026.1721481

**Published:** 2026-03-13

**Authors:** Jyrki Rasku, Ilmari Pyykkö, Martti Juhola, Esko Toppila, Jing Zou, Lenore Launerd, Kristin Siggeirsdottir, Palmi Jonsson, Howard J. Hoffman, Cuno Rasmussen, Paolo Caserotti, Vilmundur Gudnason, Hannes Petersen

**Affiliations:** 1 Department of Otorhinolaryngology, Hearing and Balance Research Unit, Tampere University, Tampere, Finland; 2 Faculty of Information Technology and Communication Sciences, Tampere University, Tampere, Finland; 3 Department of Otolaryngology-Head and Neck Surgery, Changhai Hospital, Second Military Medical University, Shanghai, China; 4 Laboratory of Epidemiology, Demography, and Biometry, National Institute on Ageing, Bethesda, MD, United States; 5 Icelandic Heart Association, Kopavogur, Iceland; 6 Department of Geriatrics, Landspitali University Hospital, Reykjavik, Iceland; 7 Faculty of Medicine, University of Iceland, Reykjavik, Iceland; 8 Epidemiology, Statistics, and Population Sciences, National Institute on Deafness and Other Communication Disorders (NIDCD), NIH, Bethesda, MD, United States; 9 Department of Sport Sciences, University of Aarhus, Aarhus, Denmark; 10 Centre for Active and Healthy Ageing, Department of Sports Science and Clinical Biomechanics, University of Southern Denmark, Odense, Denmark; 11 Department of Surgery, Akureyri Hospital, Akureyri, Iceland

**Keywords:** aging, multidimensional data vectors, posturography, sex differences, time-domain variable analysis, use of AI

## Abstract

**Objective:**

Force platforms are widely used to assess postural stability and fall risk in older adults. However, traditional parameters often capture overlapping phenomena and fail to fully reflect underlying control mechanisms. This study evaluated combining of six partly independent parameters to distinguish sex and age-related differences in postural control among community-dwelling elderly.

**Methods:**

A total of 4,588 adults aged 65–95 years were assessed using static posturography under non-visual conditions. Six time-domain parameters, reflecting torque control, positional control and anticipatory control of the center point of force. Romberg’s quotient was included for comparison.

**Results:**

Females exhibited greater stability, whereas males relied more on corrective force moments and showed larger sway amplitudes. Classification trees predicted sex with 71% accuracy using three parameters. Aging was associated with increased anteroposterior sway amplitude and a reduction in the critical time for transition between open- and closed-loop control. Additional age-sensitive parameters included mediolateral velocity zero-crossing rate and steady-phase duration. Age could be predicted within ±5 years for both sexes. Romberg’s quotient could discriminate age in 30% and sex differences in 60% of participants, only.

**Conclusion:**

Postural stability is influenced by both sex and age. The identified combination of parameters provides a framework for estimating the “biological age” of postural control and investigate balance impairments. Age-related decline appears consistent within a 5-year range, bur does not exclude the effect of lifestyle or comorbid factors. This study demonstrates that use of multidimensional data vectors with the implementation of AI-modeling can improve the predictive accuracy and clinical applicability of posturography.

## Introduction

Adequate muscle strength, efficient gait, and good balance are essential components of independence and overall wellbeing, yet these capacities decline with age (Era et al., 2006). Investigating how aging impacts parameters related to balance and strength is of critical importance for both clinicians and patients. Balance is maintained by sensory inputs from vision, the somatosensory system, and the vestibular system, which work together to detect and process positional, velocity, and acceleration information, thereby maintaining postural stability (Horak, 2006; Pyykkö et al., 1991; Enbom et al., 1991).

Human postural control is dynamic, evolving through context-dependent learning and subject to age-related changes (Abrahamova and Hlavacka, 2008). Declines in postural control typically begin around the age of 60 (Era et al., 2006), increasing susceptibility to stumbles and falls (Tinetti et al., 1994; Maki et al., 1994). Approximately 50% of individuals aged 75 years or older experience fall annually (Fuller, 2000). Fear of falling further restricts daily activity, diminishing quality of life (Tuunainen et al., 2011). The multifactorial etiology of falls (Blake et al., 1988; Cass et al., 1996; Tuunainen et al., 2013; Jäntti et al., 1995) complicates the identification of primary balance deficiencies. Postural stability is commonly evaluated using force platforms, which measure center-of-pressure movement and generate stabilograms (Nashner, 1983). Despite the widespread use of posturography for rapid screening (Era et al., 2006; Maki et al., 1994; Julienne et al., 2024), its clinical utility remains debated (Kingma et al., 2011; Pyykkö et al., 2000; Visser et al., 2008). However, individual sway characteristics depend on context (Horak, 2006; Toppila and Pyykkö, 2000), age (Hytönen et al., 1993), prior experience (Gautier et al., 2008), and overall health (Sack et al., 1993). The complexity of stabilogram-derived data can obscure meaningful insights into balance deficits (Palmieri et al., 2002; Piirtola and Era, 2006).

Haeggstrom et al. (2006) identified 163 distinct parameters used to describe human posture, underscoring the lack of consensus in measurement and analysis (Kingma et al., 2011). Previously we studied interrelations among 30 time-domain variables and showed that velocity- and amplitude-related variables are strongly associated, as are moment- and sway-area-related parameters, reflecting overlapping control mechanisms (Rasku et al., 2012).

The aim of the present study was to determine whether a selected set of stabilogram variables, recorded during quiet stance without visual input, can identify sex- and age-related differences in postural control among community-dwelling older adults. We examined the hypothesis that postural control strategies in the elderly comprise three components: (a) torque control, (b) positional control, and (c) anticipatory control that can be recorded on the force platform.

## Materials and methods

### Participants and study design

**Figure 1:**
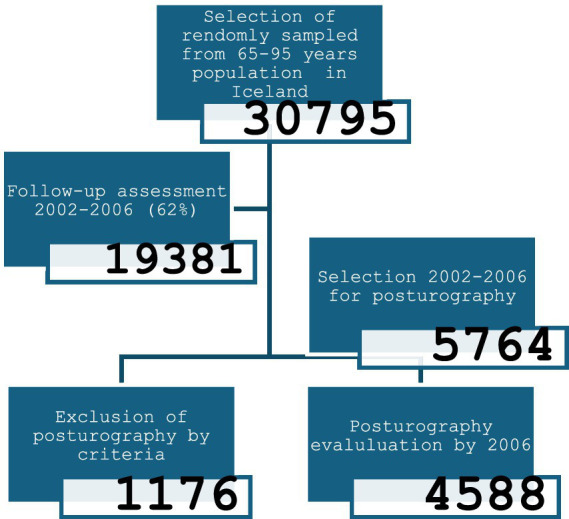
Participants in the survey and their number.

Postural stability was assessed in 4,588 elderly participants from the AGES-Reykjavik Study cohort, aged 65–95 years in connection with a larger study (Age gene/Environ-ment Susceptibility Reykjavik Study; AGES-Reykjavik). The cohort was randomly sampled from 30,795 Reykjavik residents born between 1907 and 1935. Recruitment achieved a rate of 62%, with 19,381 individuals attending the initial assessment. By 2006, 5,764 surviving members were re-examined, and 4,588 participants (2,664 females and 1,925 males; mean age 76.2 ± 5.4 years) were included in this study. Figure 1 shows the age distribution of the subjects. Participants were stratified into five age groups: 65–69, 70–74, 75–79, 80–84, and ≥85 years.

**Figure 2:**
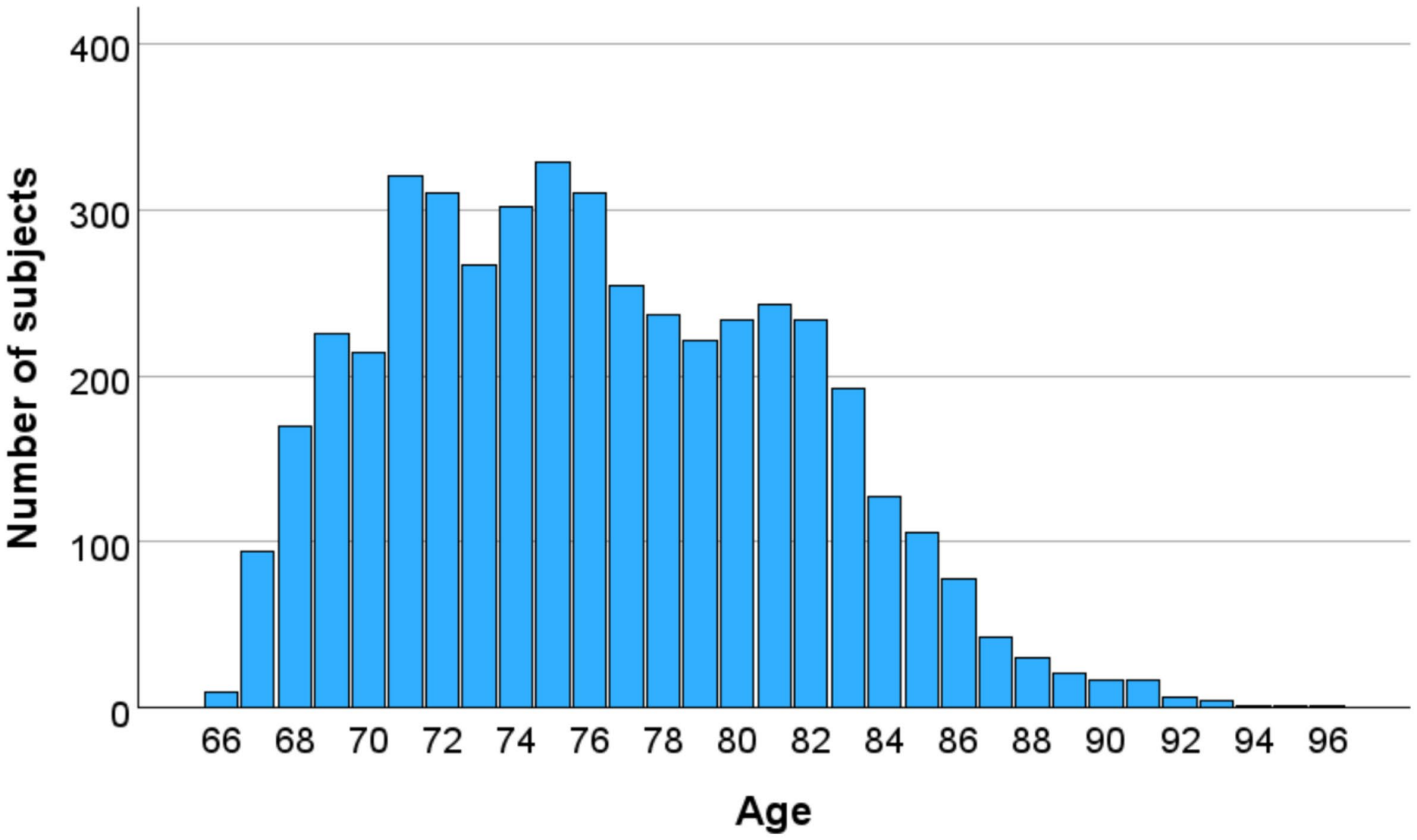
A subject standing on force platform.

Exclusion criteria included blindness, wheelchair dependency, inability to stand unaided for 30 s, or requiring assistance during a chair rise test. Ethical approval was obtained from the Icelandic National Bioethics Committee (VSN: 00–063) (Figure 2).

### Test setup

**Figure 3:**
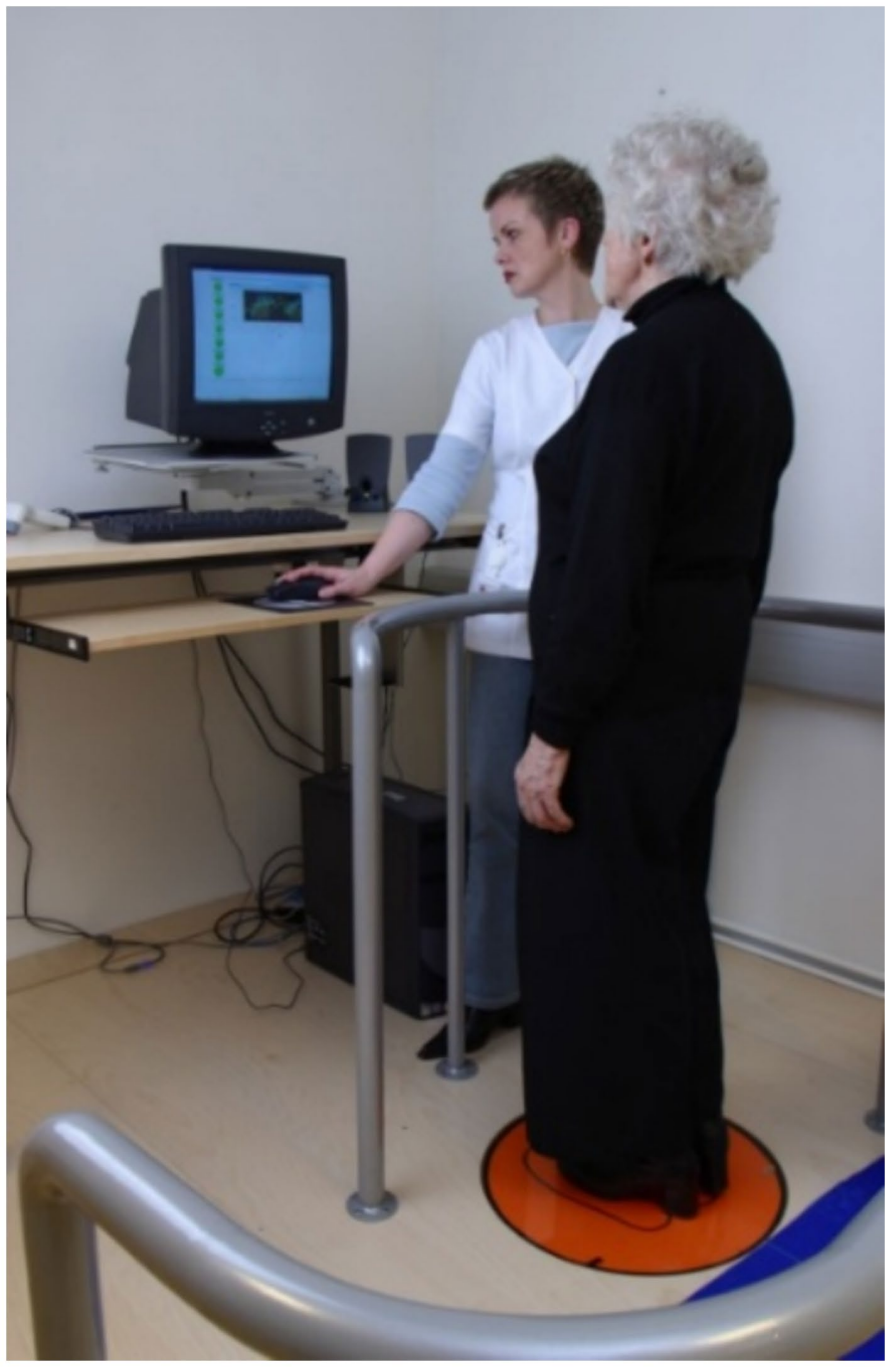
Age distribution of the subjects.

Postural stability was measured using a custom-built static force platform (Aalto et al., 1988b) that recorded the center point of force (CPF) location that calculates the changes of force.

(or pressure) on platform surface. Participants stood with arms crossed over chest (if possible), knees locked and maintained stability (Figure 3). Two conditions were tested: eyes open for 30 s and eyes closed for 30 s. Only eyes-closed conditions are reported apart of the Rombergs quotient. For referee measurements we used Romberg’s quotient to illustrate robustness of this parameter in age and sex evaluation.

### Data processing

Stabilogram data was sampled at 50 Hz, captured with medio-lateral and anterior–posterior force components and total reaction force. To remove transient artifacts the signal was filtered with median filter (Aalto et al., 1988a). Signal contamination from electrical and biological noise was mitigated using a Chebyshev finite impulse response (FIR) low-pass filter (17 Hz passband, 21 Hz stopband, maximum passband ripple 1 dB, stopband attenuation 80 dB). When subjects were placed on the force platform, they often tend to readjust their position after beginning of the measurement leading to transition error (Peterka, 2000). Preprocessing excluded the initial 15 s of data at the beginning of signal, leaving 600 samples (12 s) for analysis. Before further processing, we removed the mean values of the stabilogram’s positional components. The weight signals were normalized, altering the mean to 0 and the variance to 1. However, in the moment calculations we used the filtered weight signal without normalizing.

### Selected variables

The selection of the variables for the analysis was carried out following the procedure reported here below.

1. We first analyzed 30 different variables which suggested three different controlling strategies consisting of (1) torque control, (2) positional control and (3) anticipatory control of the center point of force (Supplementary Tables 1–3).
2. Then we analyzed correlation between the variables and found that the variables could be diminished to 17 without significant information loss (Rasku et al., 2012).
3. For these remaining variables we performed factor analysis and excluded variables that were associated with only modest loading on all factors. The threshold for the exclusion was 0.1. We also excluded variables with correlations over 0.5 as these were at least partly describing the same control strategy. In addition to the commonly used variables, we included variables that we calculated and considered important. The results of the analysis suggest that three different strategies (factors) are used to maintain an upright stance. We characterized the factors as follows: factor 1 is a torque control, factor 2 a positional control and factor 3 an anticipatory control. These 3 factors accounted for 63% of the variance in the selected variable set (Rasku et al., 2012).
4. From the final variables we further eliminated less important parameters (see Appendix) that were not associated with unique postural control strategy leaving six time-domain parameters.

In the final analysis we focused on following six time-domain variables (Pyykkö et al., 2000; Toppila and Pyykkö, 2000; Rasku et al., 2012) (Table 1).

**Table 1:** Variables selected to describe postural control in elderly.

Variable abbreviation	Variable name	Variable description/meaning
C(Y)	Amplitude range of anteroposterior sway (peak-to-peak)	C(Y) represents the range of body sway in the anteroposterior (Y) direction. A larger range indicates greater postural instability.
M(MY)	Mean moment in anteroposterior sway	M(MY) reflects neural conduction, processing, and muscle activation within a pendulum model of posture. Higher values are associated with increased muscle force activity required to maintain postural stability.
ZCR(Y)	Zero-crossing rate of anteroposterior sway	ZCR(Y) characterizes postural control responses associated with increased physical effort to regulate body sway across the neutral center point in the anteroposterior direction.
ZCR(VX)	Zero-crossing rate of mediolateral sway velocity	ZCR(VX) describes changes in CPF sway velocity across the neutral center point in the mediolateral direction. Higher values reflect postural instability and increased physical effort required to control body sway.
CRI(T)	Critical time for open- to closed-loop transition	Open-loop control relies on pre-programmed strategies independent of sensory feedback, whereas closed-loop control incorporates vestibular, visual, and somatosensory information. CRI(T) denotes the critical time point at which short-term and long-term linear fits intersect, corresponding to the transition between these control mechanisms.
ST(N)	Steady-phase standing periods	ST(N) represents local stationary periods during stance and reflects reduced postural muscle activity between transient changes in center-of-pressure behavior.

Variable C(Y). Using positional data, we calculated the peak-to-peak sway amplitudes in the anterior–posterior direction and determined the confidence limits encompassing 95% of the stabilogram samples. These metrics represent the 95% confidence amplitude projections in the anterior–posterior direction, denoted as C(Y).

Variable M(MY). Corrective movements influence the stabilogram by inducing changes in moment and force reactions due to ankle and hip torque adjustments. To quantify these effects, we calculated the mean absolute moment about the anterior–posterior axis, normalized by the subject’s body mass. This metric is referred to as M(MY).

Variables ZCR(Y) and ZCR(VX). To characterize postural force activity and quantify postural instability, we measured the frequency of CPF crossings over the stability center point. In the medio-lateral direction we calculated how many times the center point of force crosses the y-axis ZCR(X) and in antero-posterior direction ZCR(Y). We also calculated how many times the velocity changes direction in the mediolateral direction ZCR(VX). This frequency is defined as the zero-crossing rate either by sway in antero-posterior direction, denoted as ZCR(Y) or by velocity in the medio-lateral direction, denoted as ZCR(VX). Large values of zero crossings indicate that a person’s swaying amplitudes are small, and they occur around a point that remains sufficiently stationary. On contrary, in small zero crossing situations there are long swaying amplitudes or many points around which the swaying occurs.

Variable CRI(T). To assess the efficiency of postural control, we determined the critical time [CRI(T)] marking the transition from an open-loop to closed-loop control dynamics, following the method outlined by Collins and De Luca (1993, 1995). This metric has been widely used to infer the coexistence of two mechanisms during quiet stance: an open-loop mechanism operating over short time intervals, and a closed-loop mechanism dominating at longer intervals. CRI(T) determines the time when open- loop mechanism is changing to closed-loop mechanism.

**Figure 4:**
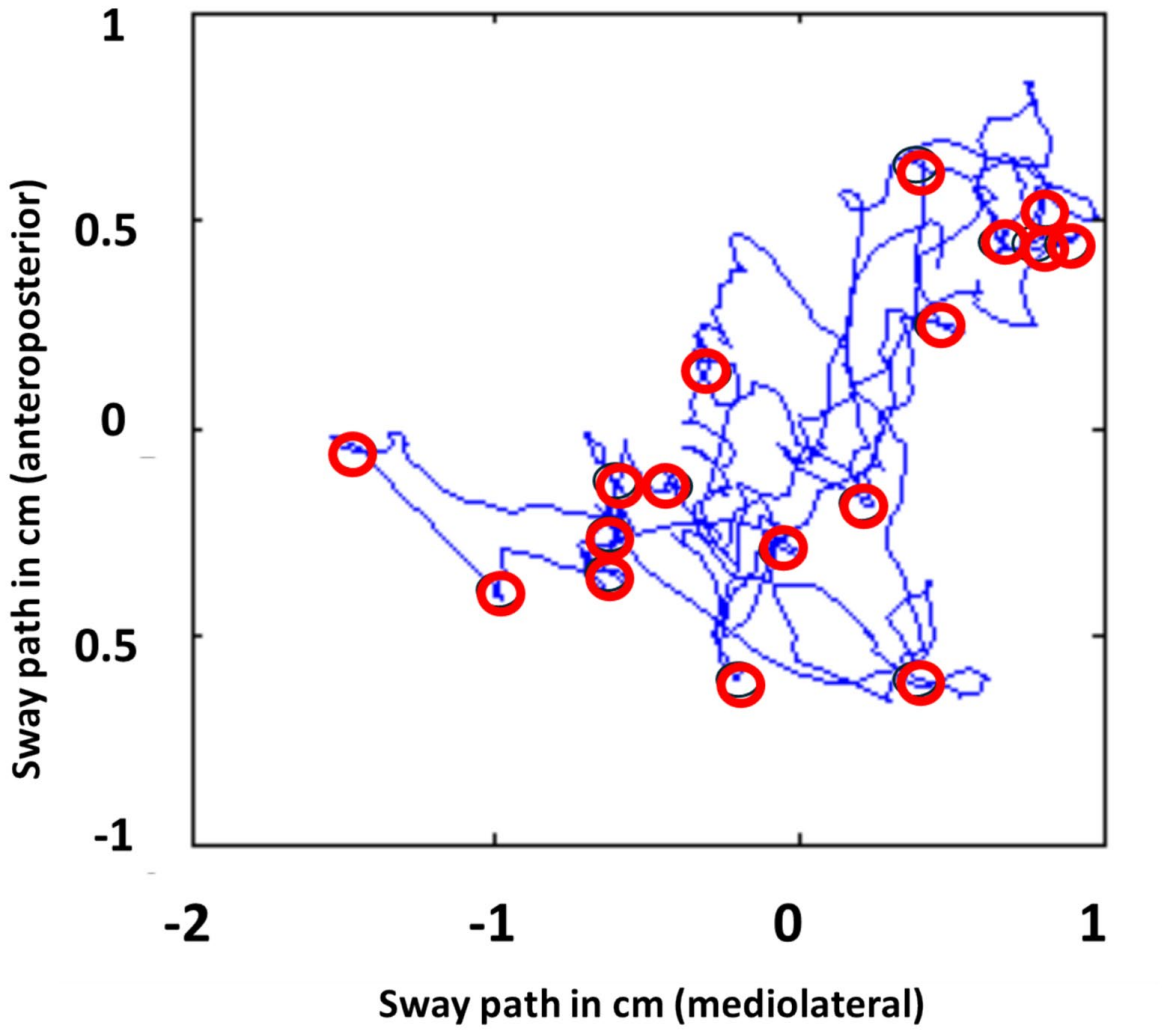
The steady-state periods (round markers) during stance on force plate posturography.

Additionally, we identified steady stance periods by calculating the number of samples corresponding to steady-phase standing intervals [ST(N)]. Figure 4 illustrates examples of presence of steady phases during quiet standing. Steady-phase standing periods [ST(N)] were identified by summing the moving variances of the moments about the medio-lateral and anterior–posterior axes. This summed moving-variance signal was normalized by dividing it by the square of the subject’s mass to mitigate mass-related influences. The threshold of low variation was set to 0.002 corresponding to the upper limit of the lowest quartile of mean value of variance of weight signal over all subjects. This value was compared to the value of the moving variance. If the moving variance was less than or equal to 0.002, the respective sample in the centered weight signal was designated as belonging to a “low variation” period.

A moment signal sample (Mi) was classified as part of a steady phase if the local variance of five consecutive moment samples, normalized by the square of the subject’s mass (Rasku et al., 2012). The local variance about a single axis at a given point (Mi) was calculated as described in formula 1. The moment reactions occur when a correction movement is made.

The 5-point moving variance centered at point i is:


$${\mathrm{Var}}_{i}=\frac{1}{5}\sum_{k=i-2}^{i+2}{\left(x_{k}-{\bar{x}}_{i}\right)}^{2}$$

Where the local mean is.


$${\bar{x}}_{i}=\frac{1}{5}\sum_{k=i-2}^{i+2}x_{k.}$$

$\bar{M}$ is the moving average of 5 moment samples, and the variance is calculated with the two preceding and two following samples of *M_i_*.

To elucidate the classical analysis of posturography the Romberg quotient (RQ) was calculated for AP-sway velocity and for peak-to peak AP sway path.

### Statistical testing and validation

For the Romberg’s quotient (RQ) analysis, we used SPSS version 26. Linear regression was applied to examine whether age or sex could be predicted from the RQ values. Finally, a decision tree model was implemented. In constructing the model, each group of 100 participants served as a training subset, and the final group of participants was used for model validation.

The data was stratified to approximate normal distributions. Natural logarithmic transformations were applied to the parameters. In data processing and statistical analysis MATLAB ver. R2014a was used. To predict sex with *decision tree* algorithm, the dataset was balanced by selecting 1,925 females whose age distribution matched that of the male participants. The male and female data were randomized and combined into a single data matrix, arranged in an alternating sequence of male and female samples. This matrix was then divided into 10 mutually exclusive subsets.

In modelling a tenfold cross-validation method was employed for *decision tree* analysis. In each iteration, nine subsets were used to train the model and estimate its parameters, while the remaining subset was used as the test set to evaluate the model’s predictive accuracy. During each round of classification, predictions were generated for 192 males and 192 females. The accuracy of sex classification was evaluated via repeated cross-validation. Cross-validation is a model validation technique used in machine learning to assess how well a model generalizes unseen data.

## Results

### Illustrating classical measures as Romberg’s quotient between sex and age groups

**Figure 5:**
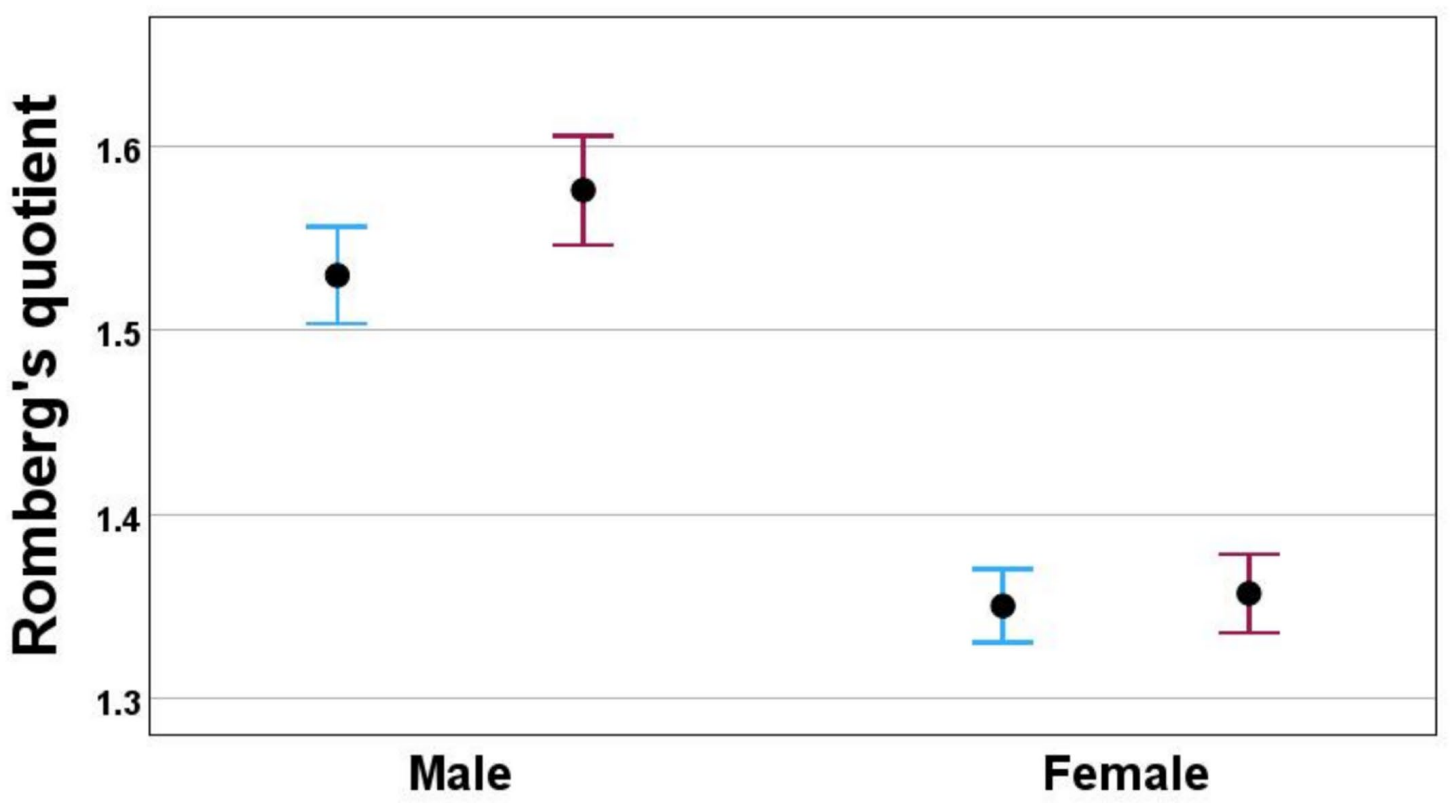
Romberg’s quotient in male and female when calculated on for antero-posterior mean velocity (dotted line) and peak-to-peak antero-posterior sway (solid line). Mean and 95% confidence intervals are shown.

The RQ was measured in peak-to-peak antero-posterior (ppAP) sway path and in antero-posterior sway velocity. The correlation between velocity and sway path was 0.618 in visual and nonvisual conditions indicating that these variables contain to great extent the same information. The measured values of visual and nonvisual condition in males and females are shown in Appendix. The females swayed less both in visual and non-visual condition than male. The sex difference in Romberg’s quotient was prominent (Mann–Whitney test *p <* 0.001 for both ppAP sway path and RQ-sway velocity). The females were less influenced by vision than males (Figure 5). There were significant differences in male between the RQ of ppAP sway path and RQ-sway velocity (Wilcoxon test, *p <* 0.001) but not in female. The RQ-sway path and sway velocity could predict the sex correctly with accuracy of 60.4 per cent (logistic regression analysis, *p <* 0.001). In decision tree analysis the sex could be predicted correctly in 14 percent of females and 92 percent of males providing overall percentage to 60 percent.

**Figure 6:**
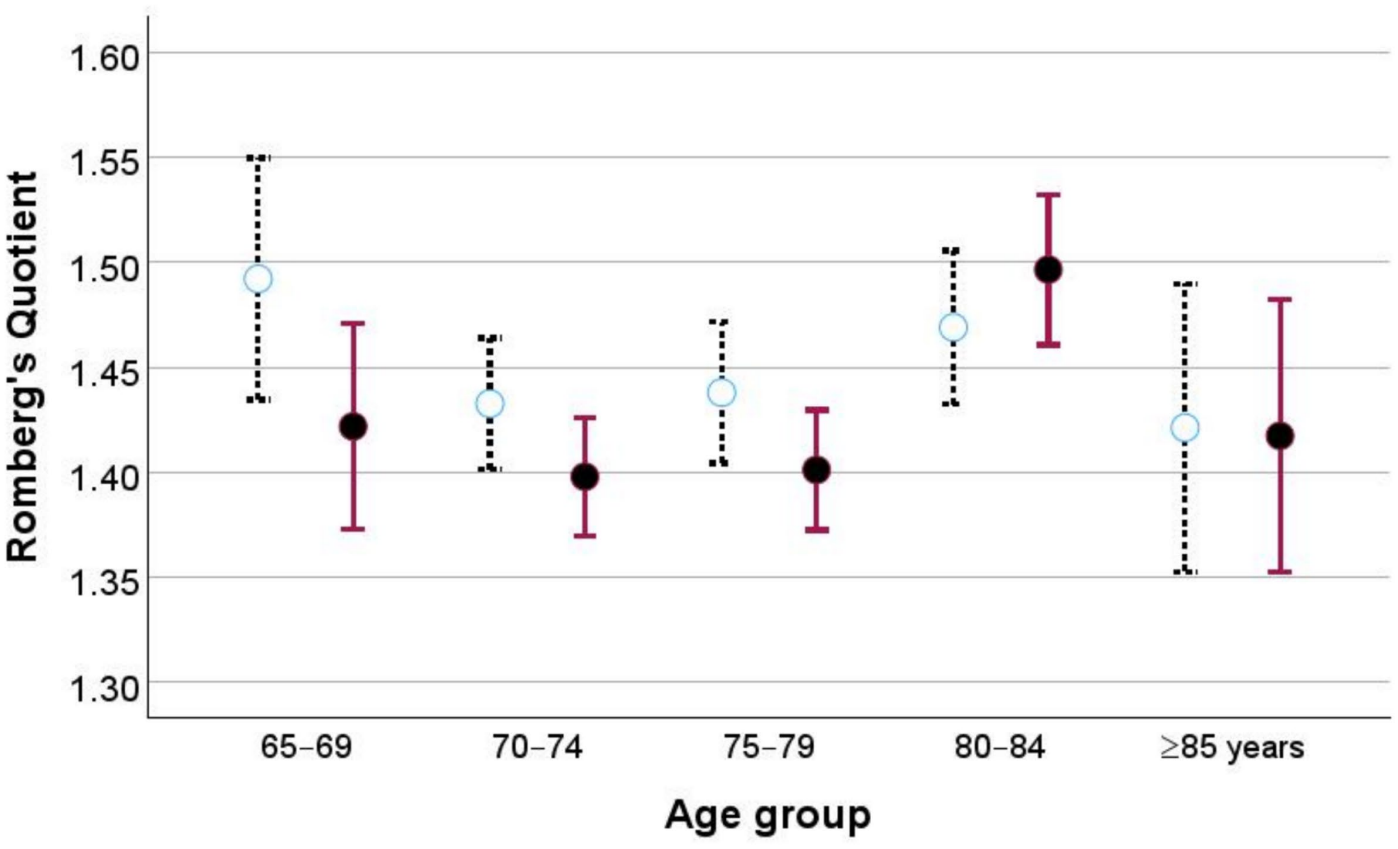
Romberg’s quotient for different age groups is presented for anteroposterior mean velocity (dotted line) and peak-to-peak antero-posterior sway (solid line). Data are expressed as mean values with 95% confidence intervals.

We observed significant differences between age groups in the RQ of ppAP sway path analysis (ANOVA, *F* = 5.73, *p <* 0.001), but not in the RQ of antero-posterior sway velocity (ANOVA, *F* = 1.40, *p* = 0.231). Pairwise comparisons revealed differences in RQ ppAP sway path and anteroposterior sway velocity analyses (Figure 6). These results highlight the statistical robustness of the models, with strong significance and moderate explanatory power.

However, substantial overlap between age groups was observed, which limits the ability to classify individuals-based age groups. In decision tree analysis the age group discrimination with accuracy of 5 years division provided an overall accuracy of 30.5 percent in both variables.

### Evaluation of sex differences with six parameters

**Figure 7:**
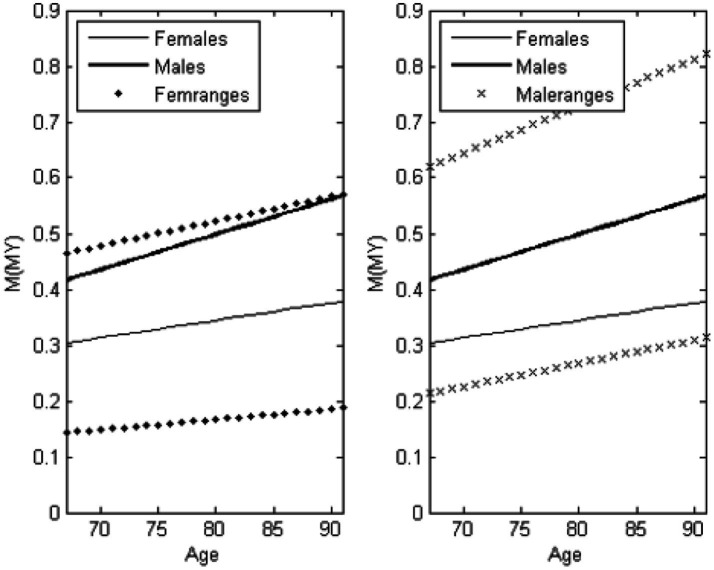
Mean values and standard deviations of the absolute moments [M(MY)] for males and females.

Table 2 shows the mean outcomes of posturography measurements for males and females. The final column (N) indicates the number of participants in each age group. These variables were selected based on best prediction of differentiation between sex and age groups. As an example, Figure 7 illustrates the linear trend of M[MY] with increasing age. The mean values are plotted separately for males and females, with standard deviations included for each sex. The plot reveals that males rarely scored below 0.2, while females seldom exceeded 0.5. This suggests that muscular strength plays a critical role in postural correction, as males generally possess greater muscular force, enabling more effective compensation for poor postural control.

**Table 2:** Mean values of the parameters for both males and females.

Age	C[Y] cm	M[MY] kg m/s	ZCR[Y] number in measuring period	ZCR[VX] number in measuring period	CRI[T] s in measuring period	ST[N] number in measuring period	*N*
Males							
≤69	0.23	0.43	14.8	87	1.03	141	176
70–74	0.30	0.47	17.7	140	0.97	81	588
75–79	0.35	0.48	18.7	131	0.91	83	590
80–84	0.40	0.49	19.7	124	0.92	82	431
≥85	0.49	0.53	22.1	123	0.83	66	140
Females							
≤69	0.23	0.32	15.7	148	1.10	225	318
70–74	0.26	0.32	17.3	165	1.10	183	809
75–79	0.29	0.33	17.7	158	1.00	182	760
80–84	0.36	0.36	19.4	145	0.93	162	594
≥85	0.41	0.36	22.1	167	0.96	146	182

C(Y), Amplitude range of the antero-posterior sway; M(MY), Mean moment in antero-posterior sway; ZCR(Y), zero crossing rate of the antero-posterior movement; ZCR(VX), zero-crossing rate of medio-lateral sway velocity; CRI(T), critical time for open and closed loop control; ST(N), Steady-phase standing periods; N, number of subjects in the respective age group.

Table 3 presents the correlation matrix of the measured parameters. The matrix was computed using a dataset that includes both male and female participants. Although the correlation coefficients showed minor sex-specific differences, the overall structure of the matrix remained consistent across groups. The abbreviations for all variables are provided in Table 1.

**Table 3:** Correlation of the selected variables.

Parameters	C(Y)	M(MY)	ZCR(Y)	ZCR(VX)	CRI(T)	ST(N)	AGE	SEX
C(Y)	1.00	0.46	0.48	0.05	−0.30	−0.58	0.32	−0.13
M(MY)	0.46	1.00	−0.01	−0.35	−0.04	−0.51	0.10	−0.33
ZCR(Y)	0.48	−0.01	1.00	0.33	−0.39	−0.37	0.18	−0.03
ZCR(VX)	0.05	−0.35	0.33	1.00	0.16	−0.11	−0.01	0.19
CRI(T)	−0.30	−0.04	−0.39	0.16	1.00	0.27	−0.11	0.08
ST(N)	−0.58	−0.51	−0.37	−0.11	0.27	1.00	−0.13	0.35
AGE	0.32	0.10	0.18	−0.01	−0.11	−0.13	1.00	−0.03
SEX	−0.13	−0.33	−0.03	0.19	0.08	0.35	−0.03	1.00

Table 4 summarizes the statistical differences in individual variables between males and females. In the indicator column, a value of 0 denotes no statistically significant difference, whereas a value of 1 indicates that the variable differs between groups (Wilcoxon test, *p <* 0.05).

**Table 4:** Statistical differences of variables between males and females in different age groups (AgeGr).

AgeGr	C(Y)	M(MY)	ZCR(Y)	ZCR(VX)	CRI(T)	ST(N)
1	0	1	0	1	0	1
2	1	1	1	1	1	1
3	1	1	1	1	1	1
4	1	1	0	1	0	1
5	1	1	0	1	1	1

0 indicates no difference and 1 difference at level of *p <* 0.05. For abbreviations of variables see Table 1.

### Evaluation of age differences

The amplitude range of anterior–posterior sway [C(Y)] was identified as the most effective parameter for predicting age. This parameter demonstrated statistically significant differences across all successive age groups for both males and females. Tables 5, 6 present the differences of variables between successive age groups among males and females.

**Table 5:** Statistical differences of variables between age groups (BwGrp) among males.

BwGrp	C(Y)	M(MY)	ZCR(Y)	ZCR(VX)	CRI(T)	ST(N)
(1–2)	1	0	1	1	1	1
(2–3)	1	0	1	0	0	0
(3–4)	1	0	0	0	0	0
(4–5)	1	1	1	0	0	1

0 indicates no difference and 1 difference at level of *p <* 0.05. For abbreviations of variables see Table 1.

**Table 6:** Statistical differences of variables between age groups (BwGrp) among females.

BwGrp	C(Y)	M(MY)	ZCR(Y)	ZCR(VX)	CRI(T)	ST(N)
(1–2)	1	0	1	1	0	1
(2–3)	1	0	0	0	1	0
(3–4)	1	1	1	1	1	1
(4–5)	1	0	1	1	0	0

0 indicates no difference and 1 difference at level of *p <* 0.05. For abbreviations of variables see Table 1.

Although not all parameters exhibited statistically significant differences between consecutive age groups, they followed consistent trends. The mean moment in anterior–posterior sway [M(MY)] increased with age, reflecting greater sway and reduced postural stability.

Clinically, M(MY) may serve as a valuable screening tool for evaluating postural compensation, particularly in cases of vestibular lesions or among frail older individuals (Tuunainen et al., 2011). This suggests that muscular strength plays a critical role in postural correction.

### Modeling of age prediction

Age prediction was conducted using data vectors comprising C(Y), ZCR(Y), CRI(T), and ST(N). For males, the standard deviation of the age prediction error was 4.9 years using linear models and 5.0 years with classification trees. Similarly, for females, the standard deviations were 5.0 years and 5.2 years, respectively.

In the linear models for predicting age for males were R^2^ = 0.152, *F* = 62.0 and *p <* 0,01, respectively. For females the respective values were R^2^ = 0.137, *F* = 75.9, and *p <* 0,01, respectively. These results highlight the statistical robustness of the models, with strong significance and moderate explanatory power.

### Modeling sex differences

**Figure 8:**
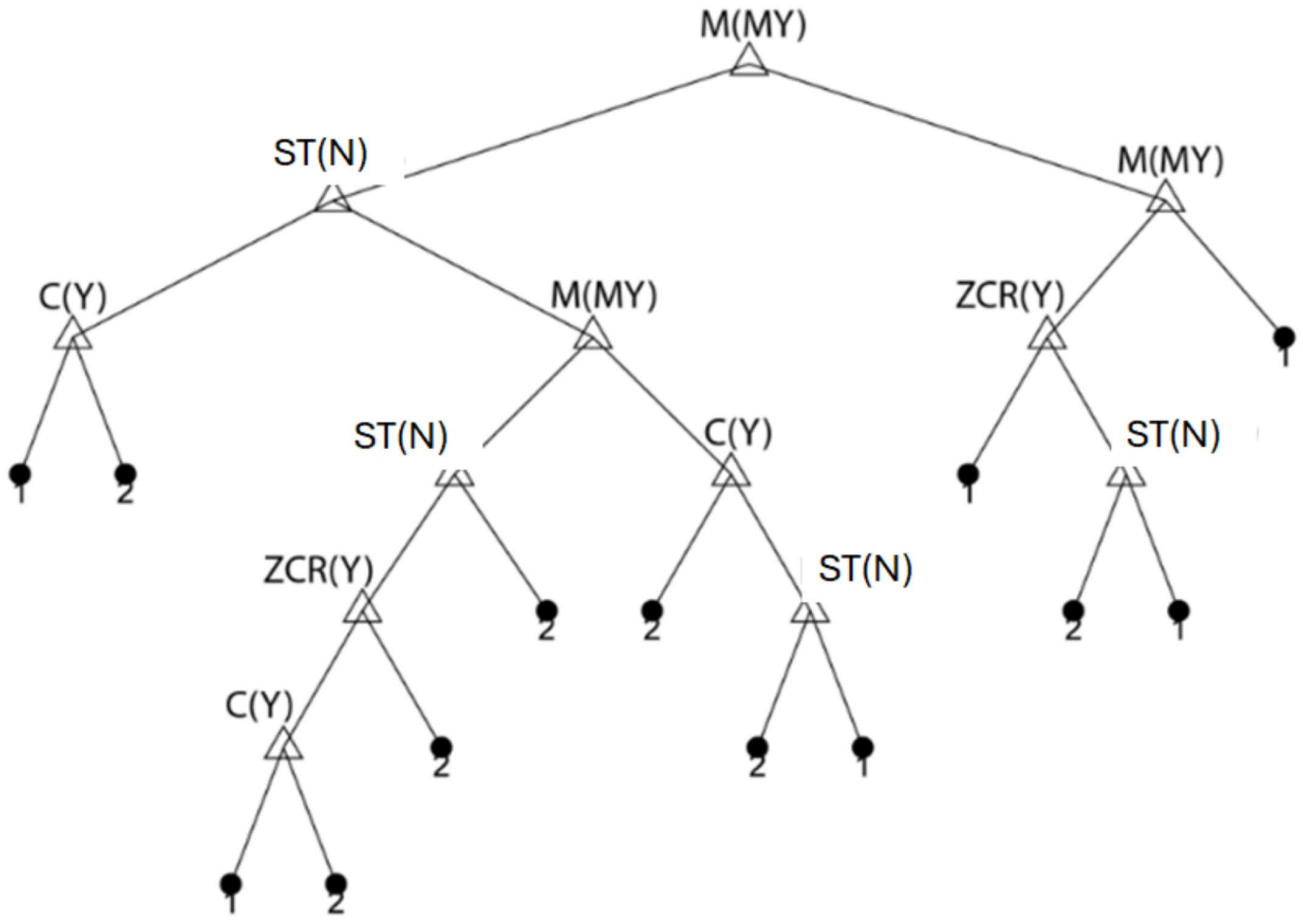
An example of a classification tree used in the prediction of sex. Leaf node 1 indicates male, and 2 indicates female. Abbreviations: C(Y) = amplitude range in the anterior–posterior direction, M(MY) = mean moment in antero-posterior sway, ZCR(Y) = zero crossing rate of antero-posterior sway, and ST(N) = steady-state period.

Figure 8 depicts the *decision tree*, where leaf nodes represent sex. The input data vectors for sex prediction included C(Y), M(MY), ZCR(VX), CRI(T), and ST(N). The classification tree achieved an overall sex recognition accuracy of 71 ± 2%, with 28 ± 4% of males misclassified as females and 29 ± 4% of females misclassified as males.

Notably, data for ZCR(VX), CRI(T), and ST(N) were excluded from the final model, indicating that these parameters were redundant for sex prediction. This suggests that sex classification is primarily influenced by other metrics such as C(Y) and M(MY), highlighting potential avenues for refining predictive models.

## Discussion

In the present study, time-domain parameters of a model comprising three distinct strategies in age- and sex-related postural stability were examined: (1) torque control, (2) positional control and (3) anticipatory control of the center point of force. The outcome of the paper demonstrates that selecting and combining variables derived from stabilogram will increase the accuracy of postrography measures that can be used for different clinical applications. To investigate age- and sex-related differences, a decision tree algorithm was applied, which is well suited for both ordinal (age) and nominal (sex) variables. Although nonlinear models may better capture the complexity of postural control, the prediction errors of linear and nonlinear approaches were comparable. Importantly, the ability to predict postural stability within a 5-year age margin suggests that age-related decline in balance is relatively consistent and difficult to evade. While individual fitness, exercise habits, comorbidities, and lifestyle factors are considered influential in delaying postural deterioration, their overall impact appears to be covered by age. This underscores the need to stimulate people to participate and tailor novel rehabilitation programs beyond conventional approaches. A more detailed analysis of posturographic data may support the design of such individualized interventions, as learning to quickly change postural strategy in defaulting sensory input.

Measuring postural stability is, however, not a simple task. In literature survey we found that 163 different parameters have been used to evaluate postural stability (Haeggstrom et al., 2006). Commonly used parameters are related to lengths, areas, velocities, and frequencies of the CPF trace (Era et al., 2006; Julienne et al., 2024; Kingma et al., 2011; Pyykkö et al., 2000). It was suggested that a subject can be selected between several control strategies that are equally efficient, and that postural control depends on the chosen strategy (Toppila and Pyykkö, 2000). To exemplify this, Peterka (2018) demonstrated that balance perturbation experiments allow estimation of “sensory weights,” which represent the relative contributions of different sensory systems to the internal estimate of body orientation used to generate corrective responses. These sensory weights are not static, but vary depending on environmental and experimental conditions, as well as neurological disorders that alter sensory input quality. Because environmental conditions may change rapidly, rehabilitation strategies should prioritize sensory reweighting. Such reweighting must occur sufficiently quickly to prevent instability resulting from under- or overproduction of corrective actions. Carroll and Freedman (1993) reported that non-stationarities may persist for up to 20 s after stepping onto the platform, which could introduce unwanted variability in short duration of posturography measurement. The authors suggested that conventional methods for parameterizing postural sway may need to be revised or reinterpreted. In the present study, stabilograms were recorded over a 12-s period. Such a short recording duration may introduce computational limitations; for example, only a few low-variance periods may be detected, indicating that the participant either stood exceptionally still or exhibited pronounced sway during the measurement. Conversely, recordings containing numerous low-variance periods also tend to include many “spiky” periods, which are indicative of large postural correction movements. The potential errors arising from variability in short recordings may be partly explained by the non-stationary nature of body sway (Carroll and Freedman, 1993), which can fluctuate over longer posturographic recording periods.

### Parameters describing ageing process

Collins and De Luca (1993) introduced stabilogram diffusion analysis, a method that provides a quantitative statistical characterization of the apparently random variations in CPF trajectories recorded during quiet upright stance in humans. This approach produces a stabilogram diffusion function (SDF), which describes the mean square CPF displacement as a function of the time interval between CPF samples.

SDFs typically exhibit a biphasic form, suggesting the involvement of two distinct control regimes: a short-term open-loop process and a longer-term closed-loop Collins and De Luca (1993) also defined the *critical point*, corresponding to the time interval and SDF value at which the short-term and long-term linear fits intersect. This metric has been widely used to infer the coexistence of two control mechanisms during quiet stance: an open-loop mechanism operating over short time intervals, and a closed-loop mechanism dominating at longer intervals.

Subsequent studies have established normative values for SDF parameters (Collins and De Luca, 1993) and have demonstrated systematic changes in these parameters as a function of visual input (Collins and De Luca, 1995; Riley et al., 1997) age (Collins et al., 1995), and pathological conditions (Treger et al., 2020). In the present study, SDF parameters were applied to estimate the *critical time of transition* between open- and closed-loop postural control [CRI(T)].

A decline in the critical time for transitioning between open- and closed-loop postural control [CRI(T)] was observed with advancing age. Open-loop control relies on pre-planned strategies independent of environmental feedback, whereas closed-loop control incorporates sensory information from the vestibular, visual, and somatosensory systems. A reduced CRI(T) reflects a diminished ability to predict prior positions, resulting in prolonged sway and an increased risk of falls. These findings are consistent with previous reports describing the loss of “recent postural position memory” (Pyykkö et al., 2000). Fear of falling may further exacerbate these effects by restricting natural sway and limiting movement.

CRI(T) analysis also indicated unstable short-term behavior, interpreted as greater sway distance before the initiation of closed-loop control. A significant group-by-condition interaction revealed that older adults exhibit a greater reliance on visual input compared with younger, healthy individuals.

Later work demonstrated that similar results can be obtained using a linear fully closed-loop system (Peterka, 2000). In this model, the body is represented as an inverted pendulum with torque applied at the ankle joints. The applied torque consists of both a random disturbance component and a control component. The control torque is subject to a time delay that reflects neural conduction, processing, and muscle activation delays. This modeling framework enables interpretation of experimentally observed stabilogram changes in terms of variations in neural controller parameters and time delays, rather than simply distinguishing between open-loop and closed-loop control. This aspect of parameter selection remains to be studied.

Two additional parameters, the zero-crossing rate of mediolateral sway velocity [ZCR(VX)] and the steady-phase standing samples [ST(N)], were strongly influenced by age. Individuals younger than 75 years demonstrated a sharp increase in ZCR(VX) accompanied by a marked decrease in ST(N), which may reflect reduced physical activity levels (Melzer et al., 2003). Beyond the age of 75, changes in these parameters became more gradual but continued to follow a declining trajectory.

Analysis of ZCR(VX) and ZCR(Y) is particularly informative, as transient features in stabilogram signals are strongly associated with postural control strategies and compensatory actions (Prieto et al., 1996). Rehabilitation programs have been shown to “refresh postural memory,” thereby reducing transients and enhancing the effective use of the stability area (Tuunainen et al., 2013). These findings highlight the importance of ST(N) and ZCR(VX) in posturography for evaluating postural control and guiding the development of targeted rehabilitation strategies.

Aging influenced postural control, with the amplitude range in the anterior–posterior direction [C(Y)] identified as the most robust predictor of age as has been indicated previously (Pyykko et al., 1990; Fujita et al., 2005; Johansson et al., 2019). The age was predicted with an accuracy of ±5 years. We refer to this amplitude control, in that long swaying amplitudes were pronounced, allowing the person to react when postural confidence limits are reached. Normalizing the logarithm of the 95% anterior–posterior sway amplitude by body mass identified that this parameter explained approximately 15% of age-related variability. Studies have linked increased body sway with higher fall risk, especially among older women (Pajala et al., 2008; Rezaei et al., 2024).

### Parameters describing sex differences

From a biomechanical perspective, maintaining posture requires the CPF to stay within the base of support. Sensory deficits in aging, such as diminished sensitivity of foot mechanoreceptors and proprioception in calf muscles, shift reliance to visual and vestibular systems (Toppila and Pyykkö, 2000; Hytönen et al., 1993). This reliance likely explains the increased sway amplitudes seen with age and the corresponding elevated fall risk (Pyykkö et al., 2000). Strategies that maximize sway range may provide additional sensory feedback but can lead to static muscle loading and fatigue. These shifts in the CPF neutral position manifest as random positional changes, reflected in parameters such as ZCR(Y), ZCR(VX), and ST(N). In this postural control, a high oscillation rate about the stationary point was the dominant characteristic of maintaining postural control. The zero crossing rates [ZCR(Y) and ZCR(VX)] and critical time CRI(T) during which an open loop control changes into a closed loop provide information about how the subject is gaining balance control. A short critical time period CRI(T) very rapidly controls excessive postural oscillations and appears to be effective strategy in balancing a human.

Consistent with prior studies, males exhibited greater sway amplitudes and moments, while females demonstrated superior stability (Julienne et al., 2024; Era et al., 2002). The mean moment in antero-posterior sway [M(MY)] and steady-phase standing periods [ST(N)] were the most predictive sex-specific parameters. These results suggest that males employ higher-moment strategies requiring greater energy and muscle activity, whereas females rely on more stable postural control mechanisms.

### Issues to be solved

In the pendulum model used to interpret balance control in posturography, several biomechanical factors influence postural performance. These can be explored by identifying variables that capture different components of the stabilogram signal, which may have clinical relevance. Important targets include fall prevention in the elderly and the characterization of postural impairment in vestibular patients. However, no consensus currently exists regarding the optimal set of parameters (Kingma et al., 2011). This lack of agreement has hindered the widespread clinical adoption of posturography.

In the present study, we selected a comprehensive set of variables that quantify different aspects of postural stability and by evaluating their predictive value on age and sex by using artificial intelligence (AI). Pennone et al. (2024) attempted to predict falls in older adults using 53 static posturography parameters combined with AI. Their prediction accuracy was limited to 60%, and the study did not report the specific decision tree branches or leaves contributing to this outcome. Cabral et al. (2020) applied multiple AI techniques to a large dataset of community-dwelling older adults (aged 60–88 years) in a cohort expected to improve predictive sensitivity. They found that static posturography did not enhance the prediction of single or recurrent falls. Sarmah et al. (2024) used neural networks with sway-path parameters and obtained similar results, although they noted that balance analysis remains valuable for the early detection of balance deficits, facilitating timely medical care and improving quality of life through rehabilitation.

We put much effort into choosing parameters describing information on postural control strategy. We assume that variables exhibiting strong internal correlations (e.g., sway path and sway velocity) reflect the same underlying phenomenon and therefore jointly provide limited additional information on postural control strategies, in contrast to metrics that are uncorrelated or only weakly correlated. However, this assumption may be questioned, as both sway velocity and sway path might potentially capture distinct aspects of postural control.

### Comment on the methods

In their Cochrane review of posturography outcomes, Alonso et al. (2012) highlighted a scarcity of anthropometric data across the 17 studies included for sex- and age-specific comparisons, comprising 5,194 participants. Only a few studies reported key variables such as height and weight, even within so-called “normative” datasets (Lelard and Ahmaidi, 2015). Similar limitations were noted in studies evaluating the effects of proprioceptive or dynamic training on postural stability in older adults (Chiari et al., 2002). In several original reports, age and sex were not analyzed, as these factors were considered non-essential to the primary outcomes.

Among anthropometric variables, height exerted the greatest influence on postural balance, both in the overall sample and when examined separately by sex. Moreover, postural balance was more strongly influenced by anthropometric factors in males than in females (Alonso et al., 2012). A crude estimate suggests that every additional 10 cm of height increases sway amplitude and velocity by approximately 5–10%, and very tall individuals may sway 15–20% faster than shorter individuals, even when their balance is otherwise normal. In contrast, weight accounts for only 1–3% of the variability in sway velocity. In the study by Wiegmann et al. (2020), the mean height difference between men and women was only 5.8 cm, suggesting that height may be a relatively minor confounder.

In the present study, height data were not available at the time of data collection and therefore could not be incorporated into standardization procedures. Height may influence sway amplitudes, velocities, and moment-related measures, but it is unlikely to affect stabilization time or estimates of critical time. Moreover, the literature remains somewhat inconsistent regarding the magnitude of age-related effects on postural stability (Alonso et al., 2012; Wiegmann et al., 2020). Based on meta-analytic evidence, Alonso et al. (2012) reported significantly greater sway in individuals aged 50–79 years compared with younger adults. Including height data might have enabled somewhat finer age-related resolution in the current analysis. However, uncertainties introduced by height are minimized when evaluating the Romberg quotient, as it is expressed as a ratio rather than an absolute measure.

We acknowledge that the exclusion criteria tend to exclude the frailest older adults, who are at the highest risk of falls and for whom balance assessment is interesting. However, apart from individuals with visual impairment, such persons are seldom exposed to balance tests due to recognized elevated fall risk by clinical condition.

In this report, we present data on eyes-closed quiet standing on a force platform, except for the Romberg quotient analysis, as our aim was to focus on the effects of ageing and sex on multiple measures of postural stability. We intend to report results from other balance-demanding tests—such as target-reaching, gait assessment, and chair-rising—in a separate work.

## Conclusion

Computerized posturography provides valuable insights into age- and sex-related deterioration of postural control. Six posturographic parameters consisting of torque, positional, and anticipatory control of body balance were used in this study to characterize stabilogram. We link these to three distinct responses of balancing acts as efforts to diminish body oscillation and promote steady balance periods, enforce dynamic correction reactions, and allow fast postural adaptation. Aging and sex were found to differentially affect specific components of postural stability. Males exhibited greater sway amplitudes and moments, while females demonstrated superior stability. The ability to predict postural stability within a 5-year age margin suggests that age-related decline in balance is relatively consistent and largely inevitable. Selection of optimal posturographic parameters, combined with the implementation of advanced modeling techniques, is essential to improve both the predictive accuracy and clinical utility of posturography. We propose that using multidimensional data vectors with AI-based decision algorithms represents a promising approach toward achieving this goal.

## Data Availability

The original contributions presented in the study are included in the article/Supplementary material, further inquiries can be directed to the corresponding author.
