# Supplementary material for: Sex and age differences of postural control in community-dwelling older adults
Source: Front Hum Neurosci. 2026 Mar 13;20:1721481. doi: 10.3389/fnhum.2026.1721481 (PMC13021594; doi:10.3389/fnhum.2026.1721481)
Supplement: Supplementary file 1 [file Data_sheet_1.docx]

Appendix Sex and age differences of postural control in community-dwelling older adults

Calculation outcome of Romberg’s (fig.1). The sway path and sway velocity are shown for male and female in figure below.

*Figure 1. Sway velocity in nonvisual condition (A) and visual condition (B) of different age groups, Sway path in nonvisual condition (c) and visual condition (d) of different age groups.*

Table 1. Parameters analyzed from stabilogram (Rasku et al. 2012)

| **Category** | **Variable** | **Meaning (Short Explanation)** |
| --- | --- | --- |
| **Sway Amplitude** | X, C(X) | Side-to-side sway and 95% limit |
|  | Y, C(Y) | Forward–backward sway and 95% limit |
| **Low Variation Sway** | Low(X) | Quiet side-to-side sway periods |
|  | Low(Y) | Quiet forward–backward sway periods |
|  | Low(T) | Total time with minimal sway |
|  | Low(N) | Number of stable periods |
| **Sway Velocity** | VX, C(VX) | Sway speed (side-to-side) and 95% limit |
|  | VY, C(VY) | Sway speed (forward–backward) and 95% limit |
| **Moment (Torque) Variables** | K(MX), K(MY) | Peakedness: how sharp or extreme corrections are |
|  | M(MX), M(MY) | Mean strength of corrective moments |
|  | STD(MX), STD(MY) | Variability of corrective moments |
| **Stabilogram Shape (2D)** | LEN | Total length of sway path |
|  | EA | Total area covered by sway |
|  | CEA | 95% confidence ellipse area |
| **Moment Error** | MERR | Overall irregularity of posture corrections (higher = poorer control) |
| **Activity Measures** | ZCR(X), ZCR(Y) | How often sway crosses center line |
|  | ZCR(VX), ZCR(VY) | How often sway velocity reverses |
|  | ZCR(W) | Zero crossings of weight signal |
|  | STD(WT) | Variation between consecutive weight-signal crossings |
